# Adult celiac disease with persistent IBS-type symptoms: a pilot study of an adjuvant FODMAP diet 

**Published:** 2021

**Authors:** Nick Trott, Anupam Rej, Sarah H. Coleman, David S. Sanders

**Affiliations:** *Academic Unit of Gastroenterology, Royal Hallamshire Hospital, Sheffield, UK*

**Keywords:** Celiac disease, Irritable bowel syndrome, Diet, Gluten-free

## Abstract

**Aim:**

This pilot study assessed the benefits of an adjuvant low FODMAP diet (LFD) in adult CD patients established on GFD who had a normal remission biopsy.

**Background:**

Patients with biopsy-proven adult celiac disease (CD) may have on-going gastrointestinal symptoms despite adherence to a gluten-free diet (GFD). Functional gut symptoms, including irritable bowel syndrome (IBS), is one cause of persistent symptoms in CD patients.

**Methods:**

Twenty-five adult CD patients who were adherent to the GFD were recruited. These patients had histologically normal villi on their remission biopsy. A specialist dietitian then offered an adjuvant LFD. Symptom response was assessed using the Gastrointestinal Symptom Rating Scale (GSRS) from baseline to follow up.

**Results:**

Of the 25 CD patients in remission with concurrent IBS, 9 did not wish to pursue the LFD, and 1 had incomplete data. Fifteen patients completed a minimum of four weeks on the LFD (mean age 44 ± 17.3; range 43.2 years; median duration of CD follow-up 7.2 years). Global relief of gut symptoms was reported by 8/15 patients (53% *p* = 0.007). Significant reductions in abdominal pain (*p *<0.01), distension (*p* < 0.02), and flatulence (*p* <0.01) were demonstrated.

**Conclusion:**

This is the first study to demonstrate that an adjunct LFD is an effective dietary treatment for concurrent IBS in adult CD patients with biopsy-confirmed remission. Such patients should be seen by a specialist dietitian to ensure nutritional adequacy and appropriate reintroduction of FODMAP-containing foods.

## Introduction

 Celiac disease (CD) is an autoimmune disorder that results from the consumption of gluten in genetically susceptible individuals (1). Most patients with CD respond to a gluten free diet (GFD) with a commensurate reduction in symptoms and, eventually, mucosal recovery. However, a subset of patients (up to 30%) suffer either primary or secondary nonresponsive disease (NRCD) and present with persisting symptoms (2). It is important that such patients are investigated to clarify the cause of their ongoing symptomatology. Clinical pathways for non-responsive patients have been published (3,4) and highlight a range of possible causes, including ongoing consumption of gluten (the most common reason), microscopic colitis, pancreatic exocrine insufficiency, as well as functional gut disorders such as irritable bowel syndrome (IBS) (5).

Celiac disease and IBS may have a partially shared pathology; mucosal inflammation, visceral hypersensitivity, dysmotility, and dysregulation of the brain-gut axis occur, to some degree, in both conditions (6–9). Similarly, both conditions have a dietary component in their treatment: GFD in CD and a low FODMAP diet (LFD) in IBS (10-12). Only two studies have assessed the role of a low FODMAP diet as an adjuvant therapy to GFD in CD patients with persistent IBS symptoms. One was an RCT (N=50) and the other an observational (N=41) study. Neither one ensured that the CD patients had a remission biopsy prior to adjuvant LFD (13, 14).

It is vital that on-going active celiac disease be excluded, as villous atrophy (VA) can mimic IBS symptoms. Furthermore, a recent systematic review demonstrated that tests for serum tTG IgA and EMA IgA levels have a low sensitivity (below 50%) in detecting persistent VA (14).

**Figure 1:**
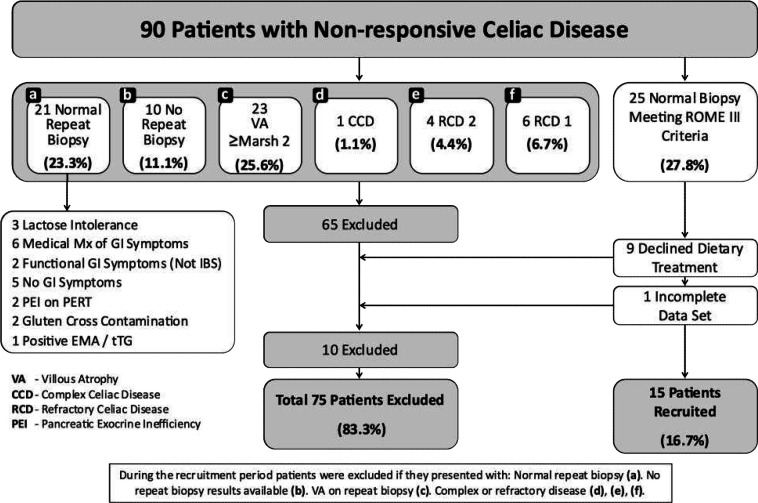
Patients recruitment 2014-2017

Although measurements of celiac serology, dietary adherence to GFD, and patient reported symptom scores are important in the follow-up of patients with CD, they are only surrogate markers of mucosal recovery (14, 15).

This pilot study assessed the benefits of an adjuvant low FODMAP diet in adult CD patients established on GFD who had a normal remission biopsy. 

## Methods

**Study Design and Participants**

In this open-label prospective pilot study, patients with NRCD were recruited from 2014 to 2017 through referrals from primary and secondary care to the dietetic service at Sheﬃeld Teaching Hospitals, United Kingdom (Figure 1). Patients who had a normal biopsy were referred by their clinician to the dietitian for an adjunct LFD (superimposed onto their gluten-free diet).

All recruited patients were older than 18 years of age and had CD deﬁned as an elevated immunoglobulin A (IgA) tissue transglutaminase (tTG) and positive IgA-endomysial antibody (EMA) in conjunction with a confirmatory duodenal biopsy on diagnosis. All patients had been following a GFD for a minimum of 2 years. 

Patients with multiple diagnoses (see Figure 1), communication barriers, positive celiac serology, or evidence of ongoing VA on biopsy were excluded.

Patients were confirmed to be in remission through negative celiac serology testing, a Marsh 1 or less on repeat duodenal biopsy, and detailed dietetic review of their current dietary intake. Concomitant diagnosis of IBS was made on the basis of ROME III criteria (16). Hematinic values (B12, folate, and ferritin) and vitamin D levels were collected at baseline and follow-up. All authors had access to the study data and reviewed and approved the final manuscript.

**Dietary advice**

All patients were assessed and followed-up by a specialist gastroenterology dietitian with experience in delivery of both gluten-free and low FODMAP approaches. Reduction of fermentable oligo-, di-, mono-saccharides and polyols (while ensuring the continued avoidance of gluten-containing products) was instigated for all participants.

Primary appointments were 60 minutes, with 30-minute follow-up appointments subsequently undertaken at a minimum of four weeks to assess the effects of the adjunct LFD. Follow-up appointments also allowed for the reintroduction of foods with higher FODMAP content according to patients’ individual tolerance. Written educational resources were provided to all patients summarizing the adjunct LFD approach. This covered foods to avoid/include recipes and strategies for navigating social situations and eating away from home.

**Questionnaire**

At both baseline and follow-up appointments, patients completed the validated Gastrointestinal Symptom Rating Scale (GSRS) (17) questionnaire that includes data on the following parameters:

- Severity of common gastrointestinal symptoms (abdominal pain, bloating, ﬂatulence, burping, borborygmi, urgency, incomplete evacuation, nausea, heartburn, acid regurgitation and lethargy) categorized as [1] None, [2] Mild, [3] Moderate, or [4] Severe (17). These individual symptom scores were also combined into a mean composite outcome score pre- and post-adjunct LFD.
- Stool consistency as classified by Bristol Stool Form Scale (BSFS) (18).
- Stool frequency was evaluated on a 7-point scale: Once a week, once every 4–6 days, once every 2–3 days, once a day, two to three times a day, four to six times a day, ≥ seven times a day.
- Gold standard global symptom question ‘Do you currently have satisfactory relief of your gut symptoms?’ (yes/no) (19).

**Statistical Analysis**

All data was analyzed using EXCEL version 16.42 (Microsoft 2020) and were summarized using descriptive statistics. Categorical data from the GSRS pre- and post-adjuvant LFD intervention was dichotomized and presented in counts and percentages. Common gastrointestinal symptoms (as listed above) were categorized as absent if patients reported them as none [1] or mild [2] and present if reported as moderate [3] or severe [4]. 

Stool form was categorized as abnormal when patients reported BSFS types one, two, six, or seven. Stool frequency was categorized as abnormal if patients reported their bowels opened less than once every 3 days or more than three times a day. This analysis strategy was replicated from Whigham et al. (2015) (20). Comparisons between categorical data were performed using Fishers exact test. Continuous demographic and serological data were compared using *t*-tests and presented with means and ± standard deviations (SD). Statistical signiﬁcance was considered when *p* <0.05.

**Ethics**

The study protocol was approved by the Yorkshire and Humber Research Ethics Committee and registered with the local research and development department of Sheﬃeld Teaching Hospital NHS Foundation Trust (REC reference 19/YH/0095). Written consent to participate was obtained from all patients. 

## Results

Between 2014 and 2017, ninety patients with NRCD were referred to our centre. Twenty-five patients met the criteria for the current study. Nine patients declined dietary treatment for their IBS, and 1 patient had an incomplete data set. Therefore, a total of 15 patients (2 males) with a median duration of follow-up of 7.2 years (range 43.2) met the inclusion criteria and completed the trial.

The demographics of all patients are outlined in Table 1. There was no diﬀerence in baseline mean age (44 ± 17.3 *vs.* 53 ± 18.4, *p* = 0.2), weight (73.6 kg ± 17.2 *vs.* 67.4 kg ± 9.4, *p* = 0.3), BMI (27 ± 5.3 *vs.* 23 ± 2.7, *p* = 0.05), baseline tTG (2.5 ± 2.0 U/mL *vs.* 1.9 ± 1.6 U/mL, *p* = 0.4), or gender (*p* = 0.3) between patients who chose to participate in the study and those who did not.

**Figure 2:**
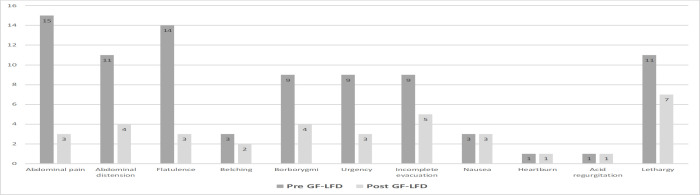
Presence of symptoms pre and post GF-LFD

Table 2 and Figure 2 show the outcomes of all patients at follow up post-instigation of the adjunct LFD. Satisfactory relief of global symptoms at follow-up was reported by 8/15 of patients (53%, *p* <0.01; Figure 1). Analysis demonstrated a significant reduction in the presence of abdominal pain (12/15, 80%; *p* <0.01), abdominal distension (11/15, 73%; *p* <0.02), and flatulence (12/15, 80%; *p* <0.01), respectively. All other mean symptom reductions were not significant. However, composite mean outcome scores at follow-up were significantly reduced (2.5 ± 0.51 vs. 1.9 ± 0.36, *p* <0.01). Mean hematinic values of ferritin (102 ± 121.1 ug/L *vs.* 100.2 ± 110.1 ug/L, *p* = 0.97), B12 (367 ± 145.5 ng/L *vs.* 537.7 ± 466.6 ug/L, *p =* 0.19), folate (10 ± 3.7 ug/L *vs.* 7.6 ± 2.0 ug/L, *p =* 0.09), and vitamin D (72 ± 27.6 nmol/L vs. 67.8 ± 33.8, *p =* 0.7) did not significantly change from baseline to follow-up.

## Discussion

**Table 1:** Demographics at baseline

Demographic	Patients Included	Patients Excluded	*p*-value
Gender: Female, n (%) Male, n (%)	13 (86.6) 2 (13.3)	7 (70) 3 (30)	0.3^a^
Age (yr), mean ± SD	44 ± 17.3	53 ± 18.4	0.2^b^
Weight (kg), mean ± SD	73.6 ± 17.2	67.4 ± 9.4	0.3^b^
BMI (kg/m^2^), mean ± SD	27 ± 5.3	23 ± 2.7	0.05^b^
Baseline tTG, mean ± SD	2.5 ± 2.0	1.9 ± 1.6	0.4^b^

BMI – Body Mass Index; tTG – Tissue Transglutaminase; ^a^ Fishers Exact Test; ^b^ Independent *t* Test

Comparison of baseline demographics of patients who completed the adjunct low FODMAP diet versus patients who declined or did not complete the dietary treatment

**Table 2:** Presence of symptoms pre- and post-GF-LFD and composite outcomes (*n*=15)

Symptoms Present, *n* (%)	Baseline	Follow Up	*p-*v*alue*
Global Symptom Question - Satisfactory Relief of Symptoms? Yes (%) ^a^	0 (0)	8 (53)	<0.01^b^
Abdominal pain	15 (100)	3 (20)	<0.01^b^
Abdominal distension	11 (73)	4 (27)	<0.02^b^
Flatulence	14 (93)	3 (20)	<0.01^b^
Belching	3 (20)	2 (13)	1^b^
Borborygmi	9 (60)	4 (27)	0.13^b^
Urgency ^c^	9 (60)	3 (20)	0.06^b^
Incomplete evacuation	9 (60)	5 (33)	0.27^b^
Nausea	3 (20)	3 (20)	1^b^
Heartburn	1 (7)	1 (7)	1^b^
Acid regurgitation	1 (7)	1 (7)	1^b^ ^a^
Lethargy	11 (73)	7 (47)	0.26^b^
Stool frequency abnormal	8 (53)	5 (33)	0.46^b^
Stool consistency abnormal	7 (47)	3 (20)	0.25^b^
Composite outcomes mean ± SD ^c^	2.5 ± 0.51	1.9 ± 0.36	<0.01^d^

^a^ Proportion of patients reporting a ‘Yes’ response to the global symptom question ‘Do you have satisfactory relief of your gut symptoms’.

^b ^Fisher Exact Test; ^c ^Composite symptom score was developed by combining all the individual symptom scores (1-4) and developing a mean score;^d^ Dependent *t *test.

This is the first prospective study to demonstrate that an adjunct LFD is an effective dietary treatment for CD patients with biopsy-confirmed remission and concomitant IBS. The gluten-free diet remains the cornerstone of treatment in celiac disease; however, it has been increasingly recognized that a subset of patients with CD (approximately 30%) present with persistent symptoms (2, 21). Recent guidelines have been published that emphasize the importance of confirming the CD diagnosis in such cases and, depending on the clinical presentation, excluding alternative causes including microscopic colitis, exocrine pancreatic insufficiency, bile acid malabsorption, and functional gut disorders, including IBS (4). As with patients who present with IBS alone, specific carbohydrate intolerances may be driving symptoms in patients with CD through changes in motility and visceral sensation (22).

When considering IBS as a parallel diagnosis, it is important to rule-out persistent villous atrophy as a cause for ongoing symptoms. While a repeat duodenal biopsy is invasive and not without risk, it is the only direct method to achieve this certainty (23).

Previous studies demonstrating the efficacy in combining gluten-free and low FODMAP approaches in NRCD have used normalization of celiac serology and dietetic review as surrogate markers for celiac disease remission (11, 12). 

However, a recent systematic review demonstrated that tests for serum tTG IgA and EMA IgA levels have a low sensitivity (below 50%) in detecting persistent VA (14). The central role of specialist dietitians in the education and support of patients with celiac disease is well established in the literature and included in national guidelines (24, 25). Dietetic review is justifiably recognized as the gold standard of assessing GFD adherence (13, 26–29); however, while one study demonstrated targeted dietetic intervention identified gluten sources and led to resolution of persistent VA in 50% of cases, the study also concluded that dietary assessment failed to identify potential gluten sources in many patients with ongoing VA (15). Equally, specialist dietetic services are underfunded and not universally available (30).

For these reasons it would seem prudent, where possible, to ensure a repeat duodenal biopsy is performed. If normal villous architecture is present, a diagnosis of associated functional bowel disease, including IBS, can be considered and dietary treatment offered, where clinically appropriate. 

In the current study, a biopsy-led approach was adopted to identify patients that may benefit from an adjunct LFD. Over 50% of the patients in the study reported satisfactory relief of their gastrointestinal symptoms on follow-up with significant reductions in abdominal pain, distension, and flatulence. This is comparable with one RCT on the use of LFD in IBS (31). In the short-term use of LFD, however, systematic reviews have indicated a significant reduction in symptoms in up to 75% of patients with IBS. One possible explanation for this disparity is that CD patients with IBS display greater symptomology, and indeed, the composite scores at baseline were higher than for other studies using the LFD for IBS alone (20).

This study does have some limitations: the small sample size and lack of control limit the study’s generalizability, and only the short-term effects of an adjunct LFD were assessed. Equally, restrictive diets such as the GFD and the LFD can be difficult to undertake and sustain. The potential treatment burden and effects on patients’ health- and food-related quality of life were not investigated. 

In conclusion, this is the first study demonstrating the efficacy of an adjunct LFD in patients with biopsy-verified CD remission and IBS. Larger trials that investigate the long-term effects of an adjunct LFD and its consequences on nutritional adequacy and food-related quality of life are warranted.

## Conflict of interests

The authors declare that they have no conflict of interest.
